# Avoiding the incompatibility of peripheral parenteral nutrition solution and midazolam injection for intravenous sedation

**DOI:** 10.20407/fmj.2020-005

**Published:** 2020-10-10

**Authors:** Akihiko Futamura, Takashi Higashiguchi, Takeshi Chihara, Yuka Yokota, Yoshinori Itani

**Affiliations:** 1 Department of Pharmacy, Fujita Health University Nanakuri Memorial Hospital, Tsu, Mie, Japan; 2 Department of Surgery and Palliative Medicine, Fujita Health University, School of Medicine, Toyoake, Aichi, Japan; 3 Department of Regulatory Science for Evaluation & Development of Pharmaceuticals & Devices, Faculty of Medical Technology, School of Medical Sciences, Fujita Health University, Toyoake, Aichi, Japan; 4 Department of Laboratory Medicine, Fujita Health University Nanakuri Memorial Hospital, Tsu, Mie, Japan

**Keywords:** Incompatibility, Midazolam, Parenteral nutrition, Sedation, Terminal cancer patient

## Abstract

**Objectives:**

We have observed white turbidity when a midazolam injection is administered from a lateral tube during the administration of a peripheral parenteral nutrition (PPN) solution. The aim of the current study was to determine how to avoid compound changes when co-administering a midazolam injection and a PPN solution.

**Methods:**

Midazolam solutions were prepared by diluting a midazolam injection with a 5% glucose intravenous infusion. We examined the formulation of the midazolam injection and a PPN solution at the concentrations used in a clinical setting for changes in appearance, pH, and midazolam content in test tubes and during administration conditions.

**Results:**

With a 1/4.8 dilution of midazolam in undiluted solution, clouding occurred. A strong correlation was revealed between the midazolam content as measured through high-performance liquid chromatography and the mixture’s midazolam concentration (R^2^=0.9918). The capture rate of midazolam infused with PPN solution was 91.0% at a 1/6 dilution, whereas it decreased to <90% at a 1/4.8 dilution.

**Conclusions:**

Our results suggest that the administration of a midazolam injection solution diluted by ≥6-fold with glucose solution or saline from a side tube during the administration of a PPN solution did not cause changes in composition.

## Introduction

Midazolam exerts a sedative effect by activating the inhibitory neurotransmitter GABA receptor in the central nervous system, and it introduces and maintains anesthesia as an injection, as intensive care sedation during artificial respiration, and as sedation during palliative care.^1^ Midazolam is the most important drug used for sedation in palliative care.^2,3^ The importance of nutrition management in palliative care has been recognized. Guidelines concerning terminal fluid therapy have been published, and peripheral parenteral nutrition (PPN) solutions are now widely used.^4,5^ A patient indicated for sedation usually receives a midazolam injection from a side tube during the simultaneous administration of a PPN solution. However, since a midazolam injection is stable under acidic conditions, the move toward alkalinity causes precipitating white turbidity or crystal precipitation. A delay in detecting this turbidity or precipitation may seriously affect a patient’s health. Clinicians must be sensitive to formulation changes because of the injection route.^6^ Although changes in midazolam injection formulations during total parenteral nutrition infusions have been reported, few reports comprehensively describe the changes occurring during simultaneous midazolam injection and PPN solution administration; only visual changes and pH observations were described, not changes in midazolam content.^7^ Therefore, we examined changes in appearance, pH, and midazolam content during administration conditions based on differences in the composition of a PPN solution and a midazolam injection solution.

## Methods

### Experiment (A)

#### 1. Samples

We often treat patients for whom a PPN solution is administered at 1000 mL/day (42 mL/h) through a main duct, and a midazolam injection (Dormicum^®^ injection solution 10 mg; Maruishi Pharm Inc.) is administered through a side line at 2 mL/h. To reproduce this situation, we mixed 8.4 mL of PPN solution and 0.4 mL of midazolam injection in a test tube at the ratio of 42:2 at room temperature and under normal light conditions. We used an amino acid and glucose injection with electrolytes and a vitamin B1 infusion (BFLUID injection; Otsuka Pharmaceutical Factory, Inc.) which is a colorless and transparent infusion agent that contains approximately 7.5% glucose, 3% amino acid (18 types), electrolytes, and vitamin B1 components (pH is approximately 6.7). We prepared midazolam solutions, (15 samples, ‘A–O’) assuming clinical doses of 10–240 mg by diluting a midazolam injection with a 5% dextrose solution (OTSUKA GLUCOSE INJECTION: Otsuka Pharmaceutical Factory, Inc.) to create a range that included an undiluted solution, i.e., 1/24 (Table 1).

#### 2. Observation of appearance changes, pH determination, HPLC

To observe changes in the appearance and pH and for high-performance liquid chromatography (HPLC), we first slowly added 0.4 mL of each sample dropwise to 8.4 mL of PPN solution, and we observed the visual changes (color tone/turbidity) immediately after the dropwise addition. After recording changes in appearance, we stirred the mixture for 30 sec using a vortex mixer and measured the pH and midazolam concentration of each sample. The pH was measured using a pH meter (F-72 pH/ION Meter, HORIBA, Ltd.). The midazolam content was analyzed through HPLC with reference to Kobo’s method,^8^ under the following conditions: column, TS Kgel ODS - 100 V (5 μm, 4.6 mm×15 cm, Tosoh Corp.); column temperature, room temperature, flow rate, 0.8 mL/min; detector, UV - 2075 Plus intelligent UV/Vis detector (JASCO Corp.); detection wavelength, 254 nm, mobile phase, 1 mM citrate-phosphate buffer (pH 5.0): methanol: acetonitrile=1:1:1 (V/V). The measurement was performed using an absolute calibration curve method using a midazolam injection solution as a standard solution. The calibration curve showed linearity in the range 3.1–50.0 μg/mL. We determined the relationship between the midazolam content as measured through HPLC and the midazolam concentration of the mixture by fitting the approximate curve. We performed a Pearson’s regression analysis to determine the correlation.

### Experiment (B)

We investigated changes in the combined PPN solution and midazolam solution, and in the combined saline solution and midazolam solution at room temperature and under normal light conditions. We prepared four midazolam solution samples by diluting a midazolam injection (Dormicum injection 10 mg) with saline to 1/2.4, 1/4.8, and 1/6, and an undiluted PPN solution (500 mL) was added dropwise via an infusion pump at 40 mL/h with a planecta infusion set filter (JMS CO., Ltd.). The 0 min time point occurred immediately after the syringe pump began to push the solution. We collected 3.5 mL of each mixed solution, having passed through the infusion in-line filter with a 0.22 μm aperture, from the tip of syringe pump over a 5 min period after 10, 30, 60, and 120 min. For the collected mixture, the pH was measured under the same conditions as in Experiment (A); the midazolam content was measured through HPLC, and the content ratio to the theoretical value derived from the standard solution was determined. We added 500 mL of saline (TERUMO, Corp) dropwise at 40 mL/h under the same conditions as with the PPN solution, and midazolam solution samples were injected from the side tube at 2 mL/hr. The dose concentration of the midazolam injection solution and the measurement conditions of the collected mixture were the same as those used for the PPN solution.

## Results

### Experiment (A)

**Figure 1:**
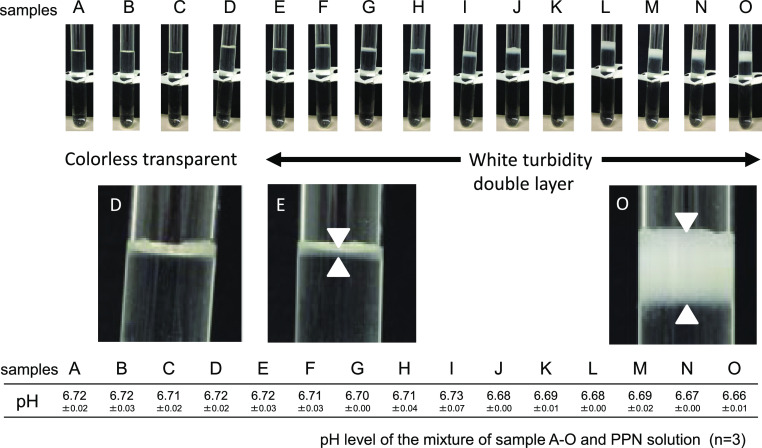
The appearance just after drop-wise sample delivery into a PPN solution. White turbidity was observed when midazolam solutions with a higher concentration than 1/4.8 diluted (1.04 mg/mL), i.e. sample E, assuming 50 mg/day administration, were mixed with a PPN solution.

**Figure 2:**
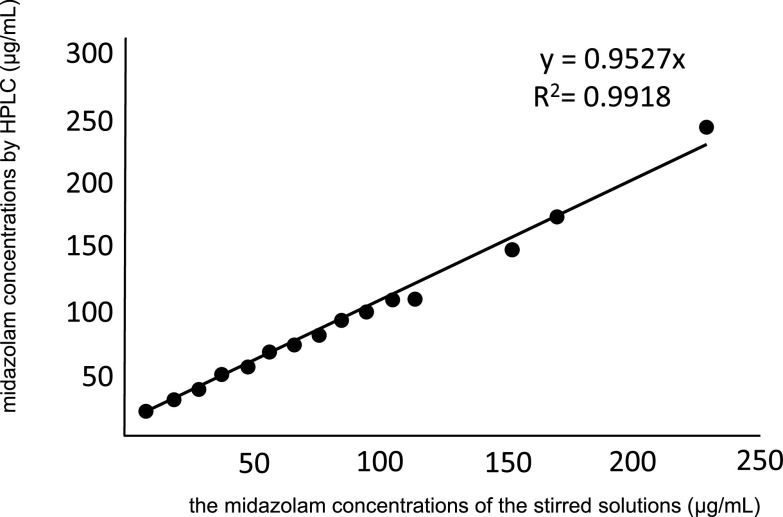
The relationship between the midazolam concentrations as assessed using HPLC and the theoretical concentrations of the stirred solutions (n=3). The coefficient of determination between the midazolam content as measured through HPLC and the midazolam concentration of the mixture was R^2^=0.9918, showing a strong correlation.

Each of the samples, from the 1/24-diluted solution of sample A to the 1/6-diluted solution of sample D, was colorless and transparent. White turbidity was observed after dropping a 1/4.8 dilution of sample E into the undiluted solution sample O. The pH of sample A was 6.72±0.02; the pH of sample E (which caused clouding) was 6.72±0.03, and the pH of sample O was 6.66±0.01 (Figure 1). The coefficient of determination between the midazolam content measured through HPLC and the mixture’s midazolam concentration was R^2^=0.9918, showing a strong correlation (Figure 2).

### Experiment (B)

**Figure 3:**
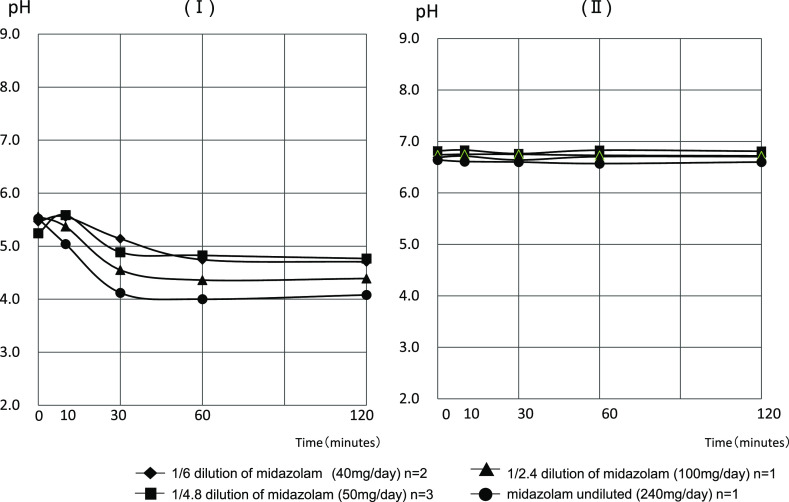
Changes in the pH of a mixed solution of saline and midazolam (I) and mixed solution of PPN solution and midazolam (II) The pH of a mixture of saline and midazolam changed to approximately pH 4 as the concentration of midazolam increased. However, the pH of the mixture with the PPN solution was approximately pH 6.7 without affecting the concentration.

The pH values of the collected mixture of PPN solution (with a pH of approximately 6.7) at 0, 10, 30, 60, and 120 min after the infusion of midazolam solution diluted to 1/6 began were 6.69±0.07, 6.72±0.08, 6.64±0.00, 6.71±0.08, and 6.71±0.08, respectively. The pH values of the mixture with saline (with pH 4.5–8.0) at the same time points were 5.47±0.04, 5.56±0.15, 5.14±0.42, 4.75±0.11, and 4.71±0.01, respectively. For the midazolam solution diluted to 1/4.8, the pH values of the mixture with the PPN solution were 6.82±0.10, 6.83±0.11, 6. 76±0.07, 6.83±0.13, and 6.81±0.11, and the pH values of the mixture with saline were 5.24±0.12, 5.59±0.08, 4.89±0.00, 4.83±0.10, and 4.77±0.10, respectively. The pH values of the mixed solution with PPN solution when injecting the midazolam solution diluted to 1/2.4 were 6.74, 6.75, 6.75, 6.73, and 6.72. The pH values of the mixed solution with saline were 5.56, 5.37, 4.55, 4.36, and 4.39. The pH values of the mixture with the PPN solution when injecting undiluted midazolam solution were 6.64, 6.61, 6.60, 6.57, and 6.60, and the pH values of the mixed solution with saline were 5.52, 5.04, 4.12, 4.00, and 4.08, respectively (Figure 3).

**Figure 4:**
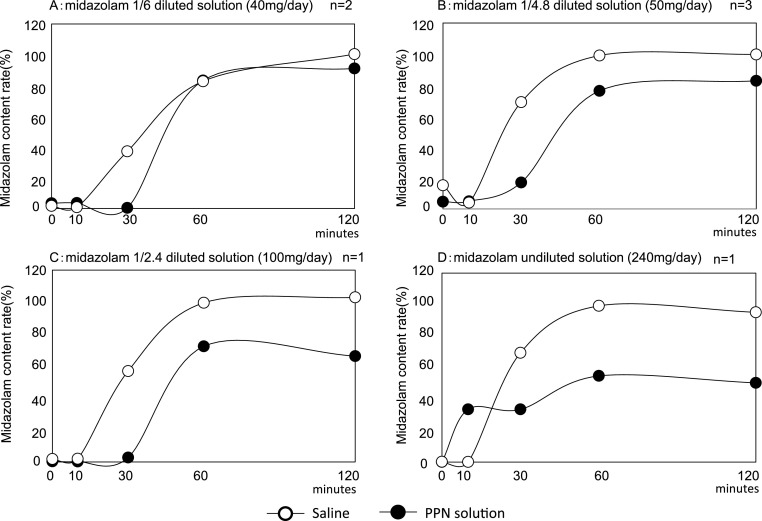
Changes in midazolam content in saline and PPN solution The content of 1/6 diluted midazolam was 90% or more after 120 min, and no change in composition occurred (4A). With the midazolam 1/4.8 diluted solution, the content of the saline mixture decreased to 99.7% after 120 min and the PPN solution mixture content decreased to 82.7% (4B). In the saline mixture 1/2.4 dilution, the stock solution showed no decrease in content even after 120 min, but the PPN solution showed a decrease in the content rate of 66.1% (4C) and 50.2% (4D).

The midazolam content ratios to theoretical values at 0, 10, 30, 60, and 120 min of the mixture with the PPN solution when injecting a 1/6 dilution of midazolam were 3.4%, 3.5%, 0.4%, 83.2%, and 91.0%, respectively. The midazolam content ratio to theoretical values of the mixture with saline were 1.6%, 0.8%, 37.1%, 82.7%, and 100.3% (Figure 4A). The midazolam content ratios to theoretical values of the mixed solution with the PPN solution were 4.6%, 4.9%, 17.0%, 76.2%, and 82.7% when a midazolam 1/4.8-diluted solution was injected, and those of the mixed solution with saline were 15.2%, 4.0%, 69.0%, 99.0%, and 99.7%, respectively (Figure 4B). The midazolam content ratio to theoretical values of the mixed solution with the PPN solution were 0.2%, 0.0%, 2.7%, 72.3%, and 66.1% when injecting 1/2.4-diluted midazolam, and the ratios of the mixed solution with saline were 1.8%, 1.9%, 56.8%, 99.5%, and 102.9%, respectively (Figure 4C).

The midazolam content ratio to theoretical values of the mixed solution with PPN solution when injecting midazolam undiluted solution were 0.1%, 33.5%, 33.4%, 54.7%, and 50.2%, and those of the mixed solution with saline were 0.1%, 0.0 %, 69.3%, 99.1%, and 95.0%, respectively (Figure 4D). The calibration curves for each concentration demonstrated R^2^=0.999 or more. An appearance change was observed only in the mix of PPN solution and midazolam undiluted solution. The mixture was colorless and transparent under other conditions.

## Discussion

Our experiments clarified a method of combined administration for a PPN solution and a midazolam injection solution that avoids changes in the mixed solution characteristics. Guidelines regarding infusion therapy for terminal cancer patients have been issued in several countries. The importance of nutrition has also been highlighted in Japan, and guidelines were issued that consider the use of PPN solution preparations.^4,9–14^ The midazolam injection solution used for the sedation of terminal-stage patients maintains a stable aqueous solution under acidic conditions, and composition changes occur under alkaline conditions. Mixing injectable drugs is not possible when one or both of the following are observed: (1) a change in the mixture’s physical appearance is recognized, (2) within 24 h post-compounding, one or more of the compounded ingredients is decomposed by ≥10%. When changes in a formulation are prevented, it remains necessary to observe the reaction product, maintain the content of the active ingredient, and ensure that the medicine is administered to the patient safely and securely.^15^

Avoiding formulation changes and reaction products and maintaining the content of the active ingredient will guarantee drug efficacy and safety. In an examination of the stability of midazolam hydrochloride in a parenteral nutrient solution with intravenous nutrients containing amino acids at 1.5%, 2.5%, and 5% concentrations and midazolam injections at 0.1 and 0.5 mg/mL concentrations, the midazolam and amino acid content at 5 h after mixing did not change, and changes in appearance, pH, and precipitation did not occur.^16^ Kuramoto et al. mixed equal volumes of a high-calorie infusion preparation and a midazolam injection solution in two-, three-, four-, and five-fold dilutions; they examined changes in appearance and conducted a filter-resistance test and reported that a five-fold dilution of the injection can be administered from the side tube of a total parenteral nutrition infusions line without causing composition changes.^7^

In Experiment (A), white turbidity was confirmed at concentrations exceeding a 1/4.8 dilution of the midazolam injection solution. Hydrochloric acid as a solubilizing agent influences changes in the midazolam formulation, leading to large variation in the clinical formulation. The pH range of midazolam injections is 2.8–3.8, and crystals precipitate at the more basic region including the change point 4.72.^17^ The most interesting point revealed in the current study is that although visible white turbidity occurred in the solution with samples E–O, when we stirred the samples again, the white turbidity disappeared and 95.27% of the midazolam could be detected through HPLC (indicating that only 4.73% was lost). These results were contrary to our expectation, because the solutions measured pH 6.6–6.7, which is more basic than the value of the changing point; i.e., the pH value at which crystals are thought to precipitate. To clarify the pH dependence of midazolam, the pH of the critical point at which no formulation change is observed could be identified using Azuma’s method or predicting a formulation change.^18^ The critical-point pH can be determined through Azuma’s method, but the reason for the value being close to the pH value of the PPN solution is that the mixed solution pH is included in the beaded infusion. The influence of the pH buffer appears to be large. Moreover, although we observed that the midazolam content of the solution after stirring does not fall, in clinical settings there is no guarantee that the mixture under the same conditions will be stirred. Therefore, midazolam administration at a ≥6-fold dilution is recommended.

In Experiment (B), the midazolam content was measured through HPLC, and we investigated whether a difference occurred in the composition change between a PPN solution transfusion and saline. We measured the pH of unbuffered saline immediately after mixing with saline and after 10 min, and the pH fluctuation after 30 min tended to be acidic due to the strongly acidic midazolam injection. This is consistent with the detection of a midazolam injection after 30 min, as shown in Figure. 4. In Figure 3 (II), the pH of a PPN solution, which has buffering properties, is shown immediately after mixing and was not affected by the pH of a midazolam injection. The change in the midazolam content over time in the saline was not detected at the mixing ratio of 42: 2 after 30 min, but was detected after 30 to 60 min. With reference to the Experiment (A) results and to confirm the reproducibility of the blending change in 1/6-diluted midazolam and a 1/4.8-diluted solution, we conducted experiments twice for the 1/6 dilution and three times for the 1/4.8 dilution. The results demonstrated that at a 1/6 dilution, the content ratio to the theoretical values of midazolam was ≥90% after 120 min from the initiation of the infusion, and no composition change occurred. With the 1/4.8-diluted solution, the midazolam content in the saline mixture decreased slightly to 99.7% after 120 min, and approximately 20% of the midazolam content was lost in the PPN solution mixture with a ratio of 82.7% because of the blending change. We speculated that the reason why the content ratio to theoretical values declined is that the trace amounts of chloride (which is colorless and transparent) were trapped by the inline filter. With the 1/2.4-diluted solution, the saline mixture after 60 min was 99.5%, and the PPN solution-mixed solution showed a deviation of approximately 30%, with a 72.3% ratio. The midazolam content ratio to theoretical values of the PPN solution-liquid mixture after 120 min demonstrated a 66.1% decrease. In the midazolam undiluted solution, the content ratio to theoretical values in the saline remained at ≥95% even after 120 min, but in the PPN solution mixture, it was 54.7% after 60 min, and approximately one-half of the expected dose was not administered. Even after 120 min, the ratio was 50.2%.

Midazolam was reported to be adsorbed by vinyl chloride, and we therefore used an infusion route with polybutadiene (non-vinyl chloride) which does not adsorb midazolam.^19^ Our results clarified that the cloudy midazolam observed in Experiment (A) is a formulation that does not reduce the midazolam content if it is redissolved. It is not yet known how the redissolved midazolam solution would affect the body. Although the cloudy component can be thought of as midazolam, an analysis of the components captured by the in-line filter is necessary because the cause of the reaction between the midazolam solution and the PPN solution that generates this white turbidity is not known.^20^ Since the injection of a high concentration of midazolam was captured by the in-line filter, it is considered that the in-line filter should be used in a clinical setting. In Experiment (B), we performed a formulation change test by setting the time for a midazolam injection to reach a patient’s blood vessel from the side tube at a maximum of 120 min. The compatibility of midazolam with morphine, haloperidol, and similar drugs which are frequently used in the palliative field should be determined through compound change tests.^21–23^

In palliative care, sedation is administered to reduce patient pain. Sedation is necessary in approximately 30% of cancer patients, for the relief of delirium, dyspnea, malaise, and pain. In a systematic review of studies of midazolam doses, the median starting dose was 0.5–1.7 mg/h, the median maintenance dose was 18–62 mg/day, and the range was 2–450 mg.^24^ Since the midazolam dose that could sedate terminal cancer patients was 20–30 mg/day, midazolam administration starts at 1–2 mg/h and the dosage is increased until sufficient sedation is achieved. Sedation using midazolam is effective at 80%–90%; at approximately 5% it causes complications leading to death, but midazolam is generally considered to be safe.^25^ Midazolam is also recommended as a palliative care sedative. Safe administration is desired; i.e., avoiding changes in formulation, complications such as pulmonary embolism, and problems such as catastrophic blockage or clogging of the in-line filter. It is important to promote the proper use of injectable medicines and to investigate and solve problems occurring with their use.

In conclusion, our findings demonstrated that the administration of a midazolam injection solution diluted by ≥6-fold or more with glucose solution or saline from the side tube of a PPN solution did not cause any change in the solution composition.

## Figures and Tables

**Table1:** Midazolam concentrations and corresponding clinical doses of the samples

Samples	A	B	C	D	E	F	G	H	I	J	K	L	M	N	O
Midazolam concentration (mg/mL)	0.21	0.42	0.63	0.83	1.04	1.25	1.46	1.67	1.88	2.08	2.29	2.50	3.33	3.75	5.00
Corresponding clinical doses of midazolam (mg/day)	10	20	30	40	50	60	70	80	90	100	110	120	160	180	240
diluted to	1/24	1/12	1/8	1/6	1/4.8	1/4	1/3.4	1/3	1/2.6	1/2.4	1/2.1	1/2	1/1.5	1/1.3	1

PPN solution is usually administered in 1000 mL/day, i.e., approximately 42 mL/hr, and midazolam solution is in 2 mL/hr using syringe pump. Therefore we mixed PPN solution and midazolam solution in the ratio of 42:2=8.4:0.4. We usually dilute midazolam solution by adding 5% dextrose solution to appropriate concentration for the patient. Therefore we prepared sample A-N by diluting midazolam injection with 5% dextrose solution. Sample O is midazolam injection itself.
